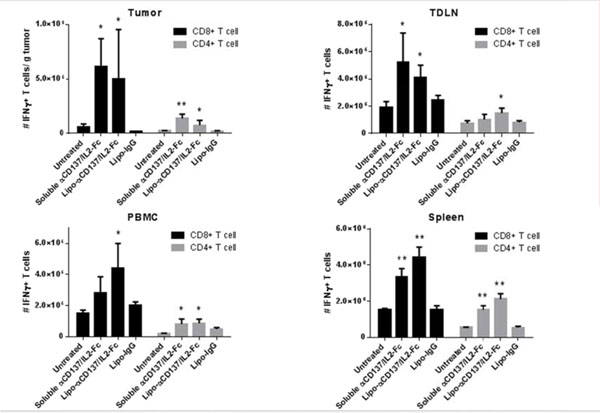# Systemic delivery of liposome-anchored anti-CD137 and IL2-Fc prevents lethal toxicity and elicits potent antitumor immunity

**DOI:** 10.1186/2051-1426-3-S2-P318

**Published:** 2015-11-04

**Authors:** Yuan Zhang, Darrell J Irvine

**Affiliations:** 1 Massachusetts Institute of Technology, Cambridge, MA, USA

## 

Many immunostimulatory drugs, such as agonistic antibodies to CD137 and interleukin (IL)-2, generate effective antitumor immune responses in preclinical studies, but demonstrate serious toxicity profiles after systemic administration, which hampers their clinical application. We recently discovered that a combination of anti-CD137 with an extended half-life IL-2-Fc fusion protein (lacking Fc receptor binding) had pronounced anti-tumor activity, but also induced toxic levels of systemic inflammatory cytokines. In order to reduce their in vivo toxicity and enhance the therapeutic window, anti-CD137 and IL2-Fc were anchored to the surface of PEGylated liposomes (Lipo-αCD137/IL2-Fc), which served as a scaffold to deliver sufficient quantities of immunostimulatory cytokines to primary and metastatic tumors following systemic administration, while minimizing systemic toxicity. In the aggressive, poorly immunogenic B16F10 melanoma model, soluble mixtures of anti-CD137 and IL2-Fc (αCD137/IL2-Fc) dramatically retarded tumor growth, but also elicited lethal toxicity in 100% of animals following 2 injections; reduced doses of this combination therapy exhibited less toxicity but also lost therapeutic efficacy. In striking contrast, mice treated with Lipo-αCD137/IL2-Fc showed significant tumor growth inhibition comparable to the soluble drug treatment, but with negligible in vivo toxicity. High levels of systemic inflammatory cytokines were triggered after intravenous administration of αCD137/IL2-Fc while minimum levels of cytokines were detected in Lipo-αCD137/IL-2-treated animals. Both αCD137/IL2-Fc and Lipo-αCD137/IL2-Fc enhanced the infiltration of cytotoxic T lymphocytes (CTL) and NK cells as well as the IFN-γ secretion in tumors. However, Lipo-CD137/IL-2 quickly cleared from the circulation after bolus IV administration and accumulated in tumor via the enhanced permeation and retention effect; while soluble αCD137 and IL2-Fc showed prolonged blood circulation profiles, which persistently activated circulating T lymphocytes, leading to toxicity and decreased survival. These results demonstrate that potent immunomodulators anchored to liposomes exhibit significantly reduced in vivo toxicity while retaining anti-tumor efficacy compared to their soluble forms at equivalent cytokine doses after systemic administration. Reduced in vivo toxicity was due to the biodistribution and pharmacokinetic profiles offered by the liposome scaffold.

**Figure 1:**
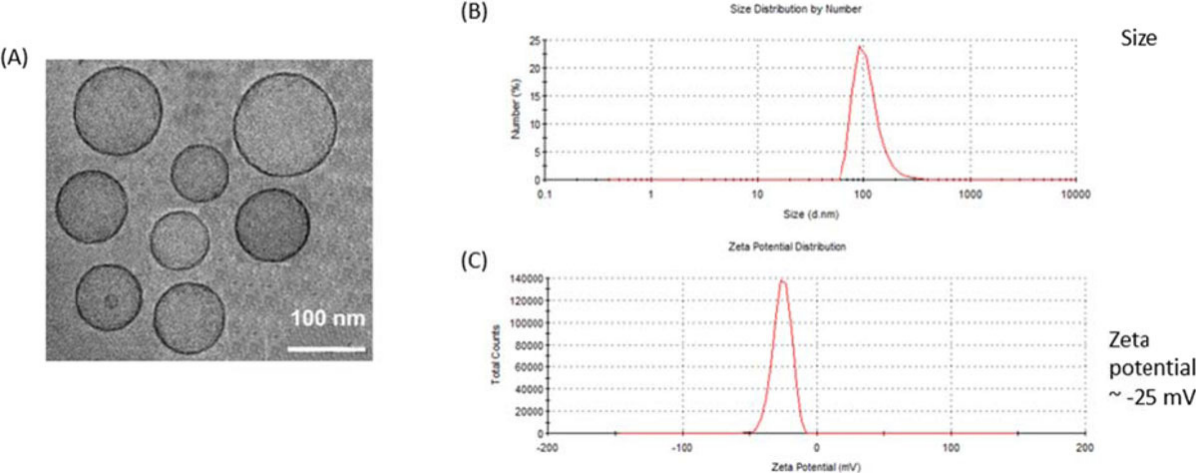


**Figure 2:**
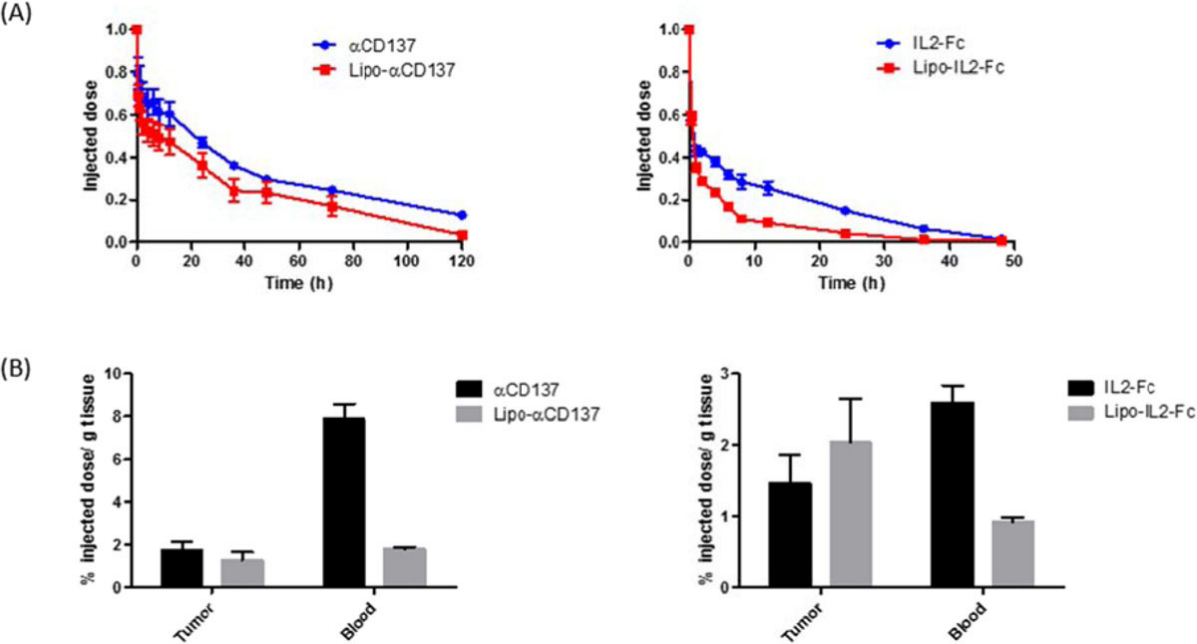


**Figure 3:**
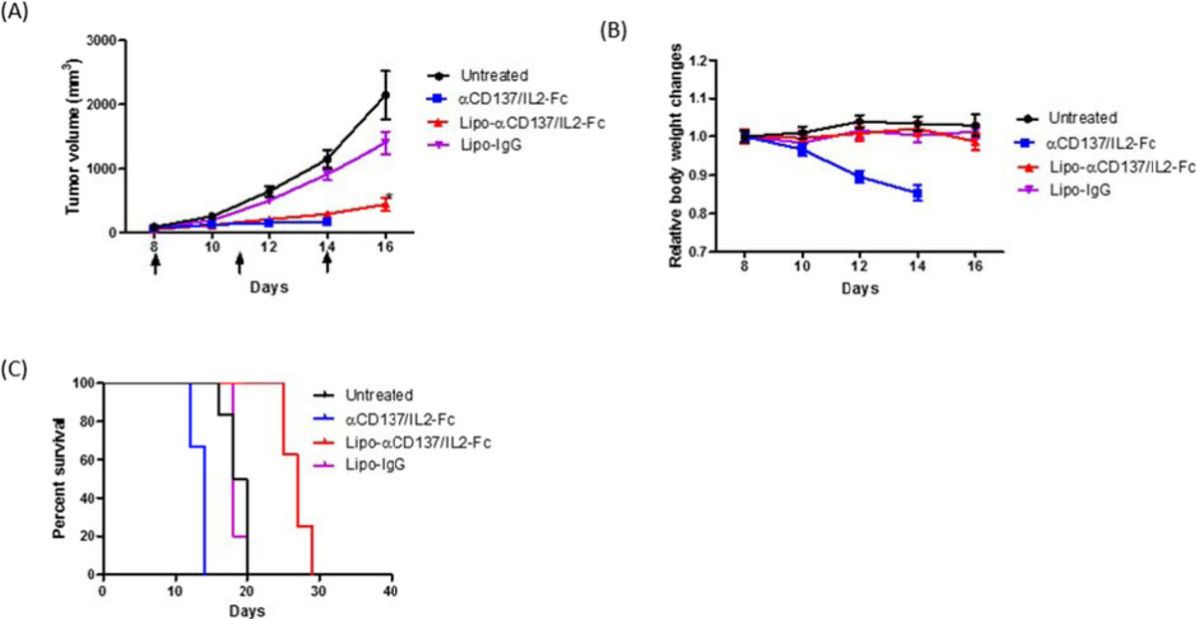


**Figure 4:**
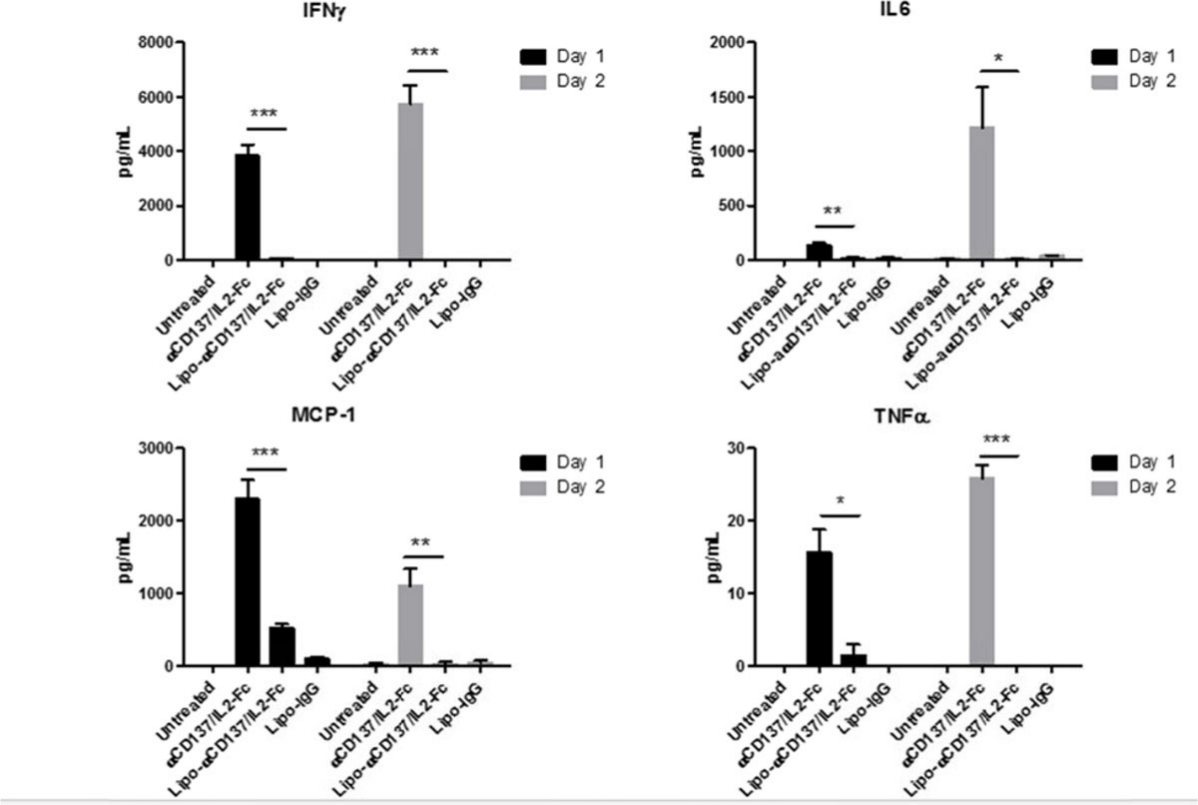


**Figure 5:**
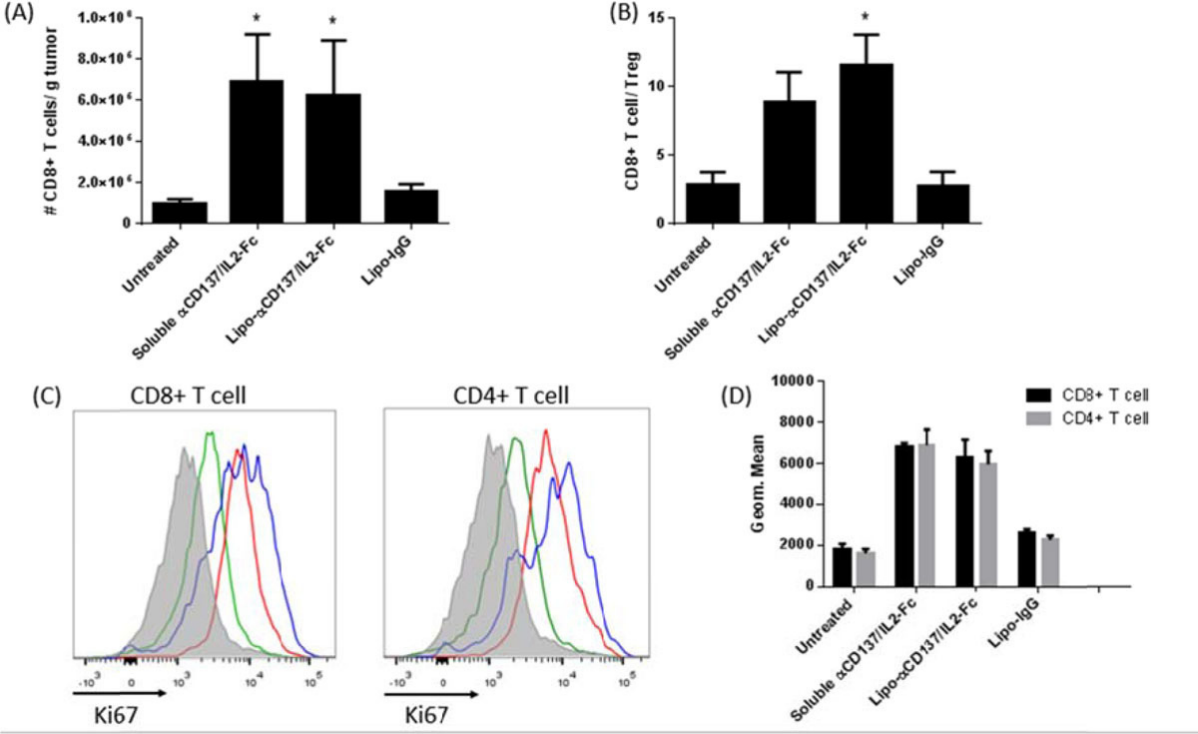


**Figure 6:**